# 

**Published:** 1916-01

**Authors:** 


### Deaths.

Dr. J. J. Shipley, of Cooke county, died at his home in Hemming on December 8th.

Dr. Forest Bedford Smith, of Houston, Texas, died December 1st. Dr. Smith had been practicing medicine in Houston for sixteen years, and was very prominent in fraternal circles.

Dr. Uriah Stone, 84 years of age, died in Quanah on December 1st.

Dr. J. S. Miller, of Mt. Pleasant, Titus county, died December 31st, after a very brief illness. The doctor was much loved, and will be greatly missed.

Dr. C. D. Cearnal, of Mumford, died November 26th.

Dr. P. R. Hengst, of Waco, Texas, died in Baltimore on December 12th.

Dr. O. S. Ewing, of Leonard, Texas, died December 11th.

Dr. Gabriel Thornhill, of Paris, Texas, died suddenly December 8th.

